# Automated and Efficient Sampling of Chemical Reaction Space

**DOI:** 10.1002/advs.202409009

**Published:** 2025-01-13

**Authors:** Minhyeok Lee, Umit V. Ucak, Jinyoung Jeong, Islambek Ashyrmamatov, Juyong Lee, Eunji Sim

**Affiliations:** ^1^ Department of Chemistry Yonsei University 50 Yonsei‐ro, Seodaemun‐gu Seoul 03722 Republic of Korea; ^2^ Research Institute of Pharmaceutical Science, College of Pharmacy Seoul National University 1 Gwanak‐ro, Gwanak‐gu Seoul 08826 Republic of Korea; ^3^ College of Pharmacy Seoul National University 1 Gwanak‐ro, Gwanak‐gu Seoul 08826 Republic of Korea; ^4^ Department of Molecular Medicine and Biopharmaceutical Sciences, Graduate School of Convergence Science and Technology Seoul National University 1 Gwanak‐ro, Gwanak‐gu Seoul 08826 Republic of Korea; ^5^ Arontier Co. 241, Gangnam‐daero, Seocho‐gu Seoul 06735 Republic of Korea

**Keywords:** chemical reaction space, dataset generation, machine learning interatomic potential

## Abstract

Machine learning interatomic potentials (MLIPs) promise quantum‐level accuracy at classical force field speeds, but their performance hinges on the quality and diversity of training data. An efficient and fully automated approach to sample chemical reaction space without relying on human intuition, addressing a critical gap in MLIP development is presented. The method combines the speed of tight‐binding calculations with selective high‐level refinement, generating diverse datasets that capture both equilibrium and reactive regions of potential energy surfaces. By employing single‐ended growing string and nudged elastic band methods, reaction pathways previously underrepresented in MLIP training sets, particularly near transition states are systematically explored. This approach yields datasets with rich structural and chemical diversity, essential for robust MLIP development. Open‐source code is provided for the entire workflow, facilitating the integration of the approach into existing MLIP development pipelines.

## Graphical abstract

The paper presents an efficient method for sampling chemical reaction space to train machine learning interatomic potentials (MLIPs). By combining fast tight‐binding calculations with selective high‐level refinement, the approach generates diverse datasets capturing both equilibrium and reactive regions. The method uses single‐ended growing string and nudged elastic band techniques to sample previously underrepresented reaction pathways, enhancing MLIPs' predictive capabilities.

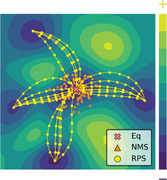

## 1 Introduction

Machine learning interatomic potentials (MLIP) are transforming molecular simulations, merging the speed of classical force fields with the accuracy of quantum methods. These models learn from quantum‐derived data to capture atomic interactions dynamically and have the potential to reveal the subtleties of bond formation and breaking—where the essence of chemistry lies.^[^ ^1^, ^2^, ^3^ ^]^ The goal of MLIPs is to replicate the potential energy surface (PES) of molecules using structural data alone, avoiding the need for expensive quantum mechanical calculations. However, the extensive nature of PES combined with the time‐consuming aspect of ab initio data collection methods presents a substantial challenge in acquiring adequate training points. An insufficient dataset for MLIPs often leads to non‐physical outputs, particularly when the model operates beyond its trained domain,^[^ ^4^, ^5^ ^]^ due to the nature of statistical learning, which does not inherently incorporate physical principles.^[^ ^6^ ^]^

**Figure 1:**
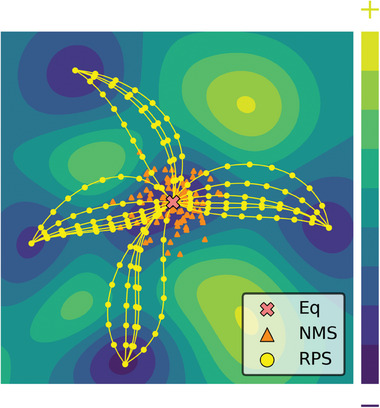
A conceptual potential energy surface with the equilibrium structure of the reactant (Eq, red “×”) at the center. Orange triangles represent structures from normal mode sampling (NMS), while yellow circles connected by pathways illustrate our reaction pathway sampling (RPS) method, which explores the potential energy surface by connecting the reactant to various products through transition states, enabling the sampling of diverse reactive pathways.

The main issue becomes effectively sampling the PES to select representative configurations. Many quantum databases like the QM series^[^ ^7^, ^8^, ^9^ ^]^ predominantly focus on equilibrium configurations, as depicted in single point “×” in **Figure** **1**, limiting MLIPs' performance in molecular dynamics simulations where molecules explore PES freely beyond equilibrium regions. Normal mode sampling, as implemented in ANI‐1^[^ ^10^, ^11^ ^]^ and QM7‐X,^[^ ^12^ ^]^ marked significant progress by generating many non‐equilibrium structures near equilibrium wells, which are illustrated by numerous triangles in Figure 1. However, its concentration on equilibrium wells still does not render MLIPs effectively predict transition states,^[^ ^13^ ^]^ underscoring the need for sampling methods that capture reactive aspects of PES to develop more accurate and reactive MLIPs.

In fact, MLIPs showed promise in simulating specific chemical reactions, such as gas‐phase reactions.^[^ ^14^, ^15^ ^]^ However, each new scenario requires a specific data set and retraining, which makes the process not only resource‐intensive but also heavily reliant on human intuition and expertise.^[^ ^16^ ^]^ To address this, Grambow et al.^[^ ^17^ ^]^ used the single‐ended growing string method (SE‐GSM)^[^ ^18^ ^]^ to generate 12 000 reactant‐product pairs from the GDB‐7 database,^[^ ^19^ ^]^ identifying a single minimum energy path for each reaction, without user‐defined products.

Furthering this work, Schreiner et al.^[^ ^13^ ^]^ applied the nudged elastic band (NEB) method^[^ ^20^, ^21^ ^]^ to these reaction pairs, incorporating often overlooked intermediate structures near reaction pathways. This approach resulted in a comprehensive 9.6 million point database, offering a more extensive exploration of the PES, particularly in reactive areas, as depicted by the circles connected by pathways in Figure 1. Integrating these two methodologies can yield a robust database for developing versatile MLIPs. However, this progress is tempered by the high computational cost associated with the reliance on density functional theory (DFT) calculations required for both the SE‐GSM and NEB methods.

**Figure 2:**
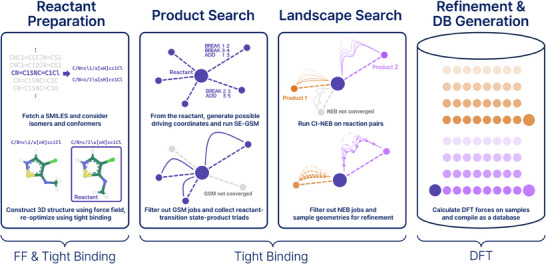
Efficient multi‐level approach for reaction pathway sampling and database generation. The protocol initiates with the preparation of reactants using the force field (FF) and computationally efficient GFN2‐xTB tight‐binding method, followed by a product search using the single‐ended growing string method (SE‐GSM) with the xTB. This step identifies potential reaction products and transition states. Subsequently, the landscape search phase explores the potential energy surface using the nudged elastic band (NEB) method, employing xTB to efficiently sample geometries along the reaction pathways. Finally, selected structures are refined using high‐level DFT calculations to generate a high‐quality database for training machine learning interatomic potentials.

Upon those foundations, we develop an approach utilizing the efficient tight‐binding method^[^ ^22^ ^]^ for sampling structures along reaction pathways, followed by selective ab initio refinement. We introduce our protocol and the associated code, designed to offer users a streamlined process for creating an extensive database that augments the capabilities of MLIPs. We demonstrate that the proposed multi‐level protocol significantly lowers computational demands while effectively capturing the essential elements of the PES. We evaluate the quality of the data generated by our method, which employs geometric graph neural network (GNN)‐based models that differ in their expressive powers for representing geometric (sub)graphs. The trained models applied as potentials can accurately describe PESs in transition state regions, particularly when combined with a normal mode sampling approach. The rest of the paper is organized as follows. In the Experimental Section, we detail the process through four stages (see **Figure** **2**): reactant preparation, product search, landscape search, and refinement with database generation. The Result section analyzes the multi‐level sampling approach, associated results, and the diversity of our generated dataset accordingly. We conclude the paper with several performance benchmarks and comparative analysis.

## 2 Experimental Section

### 2.1 Reactant Preparation

Preparing suitable reactants is essential for sampling reaction pathways and creating a robust, general‐purpose MLIP training set. The reactants were sourced from the GDB‐13 database,^[^ ^19^ ^]^ which enumerates small organic molecules containing up to 13 atoms of C, N, O, S, and Cl following chemical stability and synthetic feasibility rules. Although GDB‐13 provides a general yet chemically relevant starting point, it only includes chemical connectivity information encoded as SMILES strings,^[^ ^23^ ^]^ without explicit 3D coordinates. To generate the corresponding 3D structures and incorporate isomers, canonical SMILES strings were first created for each possible molecular structure using RDKit.^[^ ^24^ ^]^ These SMILES strings were then converted to initial 3D structures using the MMFF94 force field^[^ ^25^, ^26^ ^]^ via the gen3d functionality in OpenBabel.^[^ ^27^ ^]^ To address conformational diversity, a conformational isomer search was conducted using the Confab tool^[^ ^28^ ^]^ with the MMFF94 force field. To maintain consistency with the subsequent steps using the GFN2‐xTB method (xTB),^[^ ^22^ ^]^ all structures were re‐optimized with this method, yielding the final reactants for the reaction pathway sampling protocol.

### 2.2 Product Search

The product search phase initiates the exploration by employing the SE‐GSM to identify viable products and transition states starting from the reactant. SE‐GSM, a chain‐of‐states method, iteratively adds new structures (nodes) along a reaction pathway, beginning from the reactant and growing toward the product, while optimizing the node positions to find the minimum energy path. SE‐GSM requires driving coordinates, which are geometric parameters that define the desired structural changes along the reaction pathway, such as bond breaking or formation (e.g., ‘BREAK 1 2’ for breaking a bond between atoms 1 and 2), to guide the exploration of the PES. These driving coordinates specify only connectivity changes while allowing unrestricted exploration of all geometric features including distances, angles, and torsions along the reaction pathway. The single‐ended nature of SE‐GSM is particularly useful for exploring reaction pathways when the product structure is unknown. It can identify multiple possible products and transition states without prior knowledge of the endpoint.

The generation of driving coordinates was automated for the reactant by modifying the original codes by Grambow et al.^[^ ^17^ ^]^ This automation is achieved through a graph enumeration algorithm that utilizes predefined bond alteration parameters, such as the number of bond changes. Automating the generation of all driving coordinates is crucial for creating a general‐purpose database. It ensures a systematic and unbiased exploration of PES without relying on human intuition or prior knowledge about the system. Although the number of possible bond‐breaking and bond‐forming combinations grows exponentially with molecular size, the use of xTB makes this approach computationally feasible even for larger systems. In this work, the method was demonstrated using molecules with up to 7 heavy atoms. While the current implementation focuses on unimolecular reactions, the SE‐GSM method captures a variety of chemical transformations beyond simple fragmentations. These include intramolecular rearrangements, isomerization processes, and ring formations. (Figure S1, Supporting Information) Furthermore, since complete reaction pathways are sampled, the dataset inherently encompasses bimolecular formation reactions when the reaction paths are considered in reverse. After applying SE‐GSM, a filtering algorithm is employed to exclude trivial pathways with strictly uphill energy trajectories, negligible energy variations, and unfeasible or repetitive structures. The surviving reactant, product, and transition state triads are then compiled for the landscape search phase.

### 2.3 Landscape Search

In the landscape search phase, PES was explored using the NEB method utilizing the reactant, product, and transition state triads obtained from the product search phase. The NEB method creates a series of intermediate structures (called “images”) along a reaction pathway, connecting the reactant and product states. These images are optimized to find the minimum energy path while maintaining equal spacing between neighboring images by applying spring forces. The initial set of NEB images is generated through interpolation,^[^ ^29^ ^]^ informed by the triads from the product search phase. The minimum energy path was then optimized using NEB and climbing‐image NEB (CI‐NEB)^[^ ^21^ ^]^ iterations, adhering to convergence criteria based on the maximum force (*F*_max_) within a limited number of steps.

Standard NEB analyses typically focus on the final converged minimum energy path, representing the most energetically favorable reaction pathway. However, the protocol diverges from this approach by integrating intermediate paths encountered during the optimization process, as suggested by Schreiner et al.^[^ ^13^ ^]^ This enriched approach provides a multifaceted view of possible transformations from each reactant to various products, significantly enhancing the diversity of the dataset. By including the final converged minimum energy path and the intermediate NEB bands that lie near the reactive regions of the PES, a broader range of chemically relevant structures were captured.

Stringent criteria were applied when filtering the NEB data to ensure the data quality and relevance. Non‐convergent reactions and those with transition states lacking a single negative eigenvalue in their Hessian matrices are filtered out, ensuring a focus on structures near valid paths. Furthermore, NEB results were selected based on a criterion that prevents the redundancy of similar structural data. A new band was sampled only when the cumulative sum of *F*_max_ surpasses 0.1 eV Å ^−1^ since the last inclusion. This approach saves computational resources in the database generation phase and avoids overfitting to narrow regions of the PES. The filtered final structures underwent a high‐level refinement, ensuring the generation of a diverse and high‐quality dataset for MLIP training.

### 2.4 Refinement with Database Generation

In this phase, all the labels of the explored structures (energies and forces) were refined using DFT with the ωB97X functional^[^ ^30^ ^]^ and the 6‐31G(d) basis set^[^ ^31^ ^]^ in a way compatible with established databases like ANI1‐x^[^ ^32^ ^]^ and Transition1x.^[^ ^13^ ^]^ The protocol efficiently generated an average of ten distinct products from a typical reactant containing six non‐hydrogen atoms. 1000 data points were sampled for each product path set, augmenting the database by ≈10 000 structures for every reactant. The resulting database is encoded in hierarchical data format,^[^ ^33^ ^]^ which allows for efficient storage and retrieval of large datasets. The data is grouped by chemical formula with reaction‐specific subgroups; within these, tensors for atomic numbers, energies, forces, and positions are stored. This structured organization prepares the database for seamless integration into MLIP training sets.

**Figure 3:**
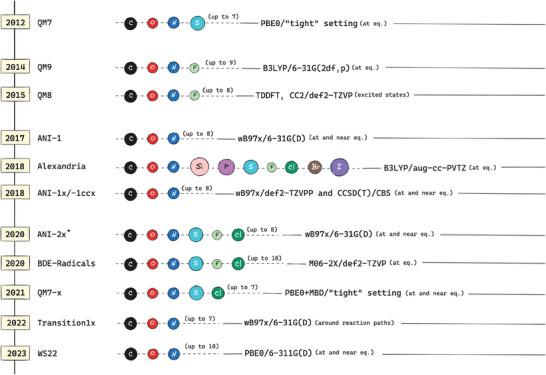
Overview of synthetic quantum databases in organic compounds. The chronological order, QM methods used, coverage and diversity of the QM‐series^[^ ^7^, ^8^, ^9^, ^12^ ^]^ and ANI‐series^[^ ^11^, ^32^, ^34^, ^35^ ^]^ along with the Alexandria,^[^ ^36^ ^]^ BDE‐Radicals,^[^ ^37^ ^]^ Transition1x,^[^ ^13^ ^]^ and WS22^[^ ^38^ ^]^ databases are illustrated. ANI‐2x database is not publicly available.

Allured by the success of MLIPs in chemistry, numerous databases have been generated over recent years. These databases all encode quantum information which is vital for the accurate modeling of interatomic potentials. **Figure** **3** provides a visual summary of the synthetic quantum databases of organic compounds. Along with the well‐documented QM series (QM7,^[^ ^7^ ^]^ QM8,^[^ ^8^ ^]^ QM9,^[^ ^9^ ^]^ QM7‐X^[^ ^12^ ^]^), and ANI series (ANI‐1,^[^ ^10^ ^]^ ANI‐1x,^[^ ^32^ ^]^ ANI‐1ccx,^[^ ^34^ ^]^ ANI‐2x^[^ ^35^ ^]^), the figure also features notable databases such as Alexandria,^[^ ^36^ ^]^ BDE‐Radicals,^[^ ^37^ ^]^ Transition1x,^[^ ^13^ ^]^ and WS22.^[^ ^38^ ^]^ The illustration emphasizes the evolution, coverage, and diversity of these databases. Each database is characterized by the elements it covers, the number of atoms, and the specific computational methods used. In particular, Transition1x excelled in capturing reaction pathways near transition states. Our work extends this approach by introducing an efficient sampling method for these critical regions, facilitating the development of MLIPs that accurately model chemical reactivity across diverse systems. The work aligns with recent efforts by Kim, Kuwahara, and Sumiya^[^ ^39^, ^40^, ^41^ ^]^ to enhance the exploration and understanding of chemical reaction spaces.

## 3 Results and Discussion

### 3.1 Multi‐Level Sampling Approach

Obtaining structures and calibrating properties with low‐level theory are standard practices in computational chemistry,^[^ ^42^ ^]^ extensively used to build chemical databases for MLIPs such as DimeNet,^[^ ^43^ ^]^ QM7‐X,^[^ ^12^ ^]^ and QMugs.^[^ ^44^ ^]^ Our protocol prioritizes computational efficiency while maintaining the ability to explore reaction pathways effectively. **Table** **1** demonstrates the stark efficiency contrast between DFT‐only and our multi‐level approach for a test reactant C_4_H_4_ClNO with 231 initial driving coordinates. In SE‐GSM calculations, our approach achieves comparable success rates (25/231 vs 28/231) while reducing computation time from 356.9 to 0.4 h. Similarly, NEB calculations maintain the same success rates (7 pathways) while decreasing computation time from 146.8 to 0.01 h. Even including the final DFT refinement (4.2 h), our total computational cost is just 4.6 h compared to 503.8 h for the DFT‐only approach—a 110‐fold speedup. This dramatic reduction in computational cost stems from xTB being ≈3000 times faster than ωB97X/6‐31G(d) calculations. Our multi‐level strategy is particularly advantageous considering that many SE‐GSM and NEB calculations produce non‐converging paths, which would waste substantial resources with high‐level methods. Using xTB for initial sampling mitigates this issue by allowing low‐cost discarding of non‐converging results followed by selective refinement, thus enabling efficient exploration of reaction pathways for MLIP training.

**Table 1:** Computational costs and success rates comparison between DFT‐only and multi‐level approaches for a test reactant C_4_H_4_ClNO with 231 initial driving coordinates. All calculations were performed using 32 processors (two Intel(R) Xeon(R) Gold 6242 CPU @ 2.80GHz).

	**DFT‐only**	**Multi‐level**
**SE‐GSM**		
Success/Total	28/231	25/231
Time (hours)	356.9	0.4
**NEB**		
Success/Total	7/28	7/25
Time (hours)	146.8	0.01
**DFT Refinement**		
Time (hours)	—	4.2
**Total Time (hours)**	503.8	4.6

### 3.2 Diversity of the Dataset

To validate our proposed reaction pathway sampling (RPS) scheme, we constructed a model dataset starting from 85 equilibrium structures, comprising molecules with up to 4 heavy atoms (C, N, O) and those with 5–7 heavy atoms (including chlorine or sulfur). The RPS method generated 332 370 structures, while we created an NMS dataset (85 085 structures) using QM7‐X^[^ ^12^ ^]^ methodology by perturbing 1000 structures per equilibrium structure along normal modes according to the Boltzmann distribution at 1500 K (see Table S1, Supporting Information for complete dataset size distributions).

**Figure 4:**
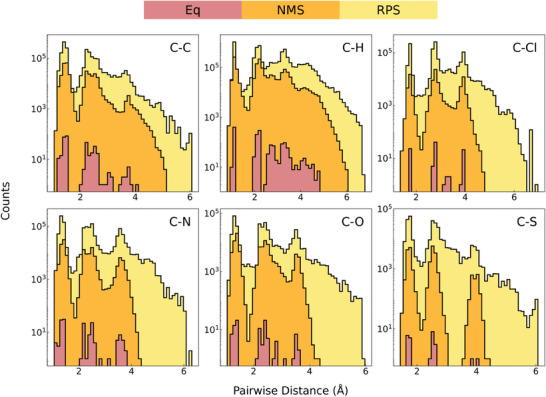
Enhanced structural diversity achieved by the reaction pathway sampling (RPS, yellow) method compared to normal mode sampling (NMS, orange) datasets, as illustrated by the pairwise distance plots. NMS and RPS datasets originate from the same 85 equilibrium structures of reactants (Eq, red), comprising molecules with up to 4 heavy atoms (C, N, O) and those with 5–7 heavy atoms (including chlorine or sulfur).

The diversity of the RPS dataset is evident in its structural coverage, illustrated by the pairwise distance plots in **Figure** **4**. The RPS method captures structures with pairwise distances exceeding 5 Å, indicative of bond dissociation, which is absent in the NMS dataset. Furthermore, within the sub‐5 Å range, RPS explores previously unsampled regions, thus creating a more comprehensive dataset for MLIP training. Notably, our analysis of triplet angles and quartet dihedral angles further (Figures S2 and S3, Supporting Information) demonstrates that the RPS method effectively samples diverse bond angles and torsional configurations, capturing critical conformational changes that occur during chemical reactions.

**Figure 5:**
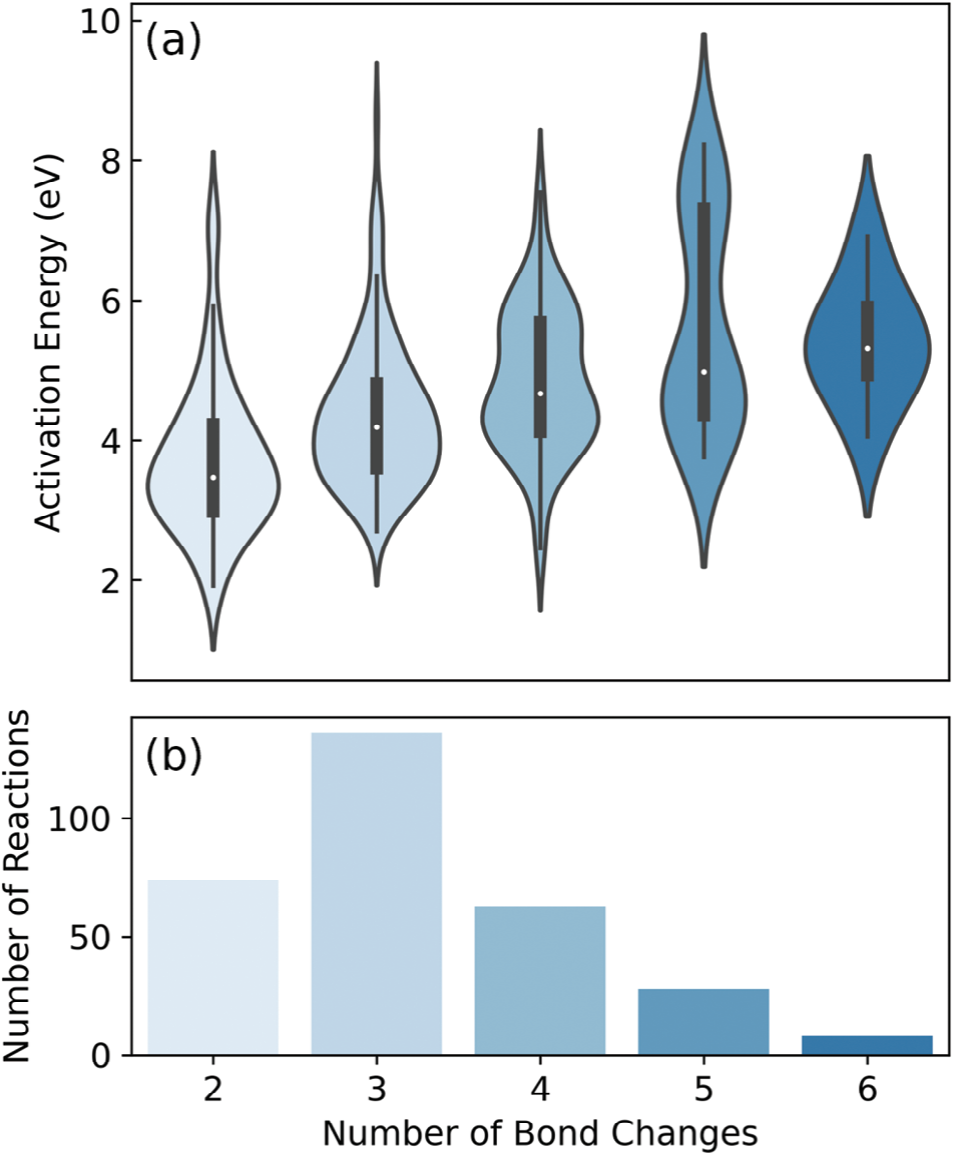
Reaction diversity captured by the RPS dataset, showcasing its ability to sample high‐energy transition states crucial for exploring reactive regions of the potential energy surface. a) Activation energy distributions split by the number of bond changes in reactions. b) Distribution of reactions across different numbers of bond changes.

**Figure** **5** illustrates the reaction diversity of the RPS dataset. This highlights its effectiveness in sampling high‐energy transition states, which is essential for exploring the reactive regions of the PES. The activation energy distribution shows that the median activation energy increases with the number of bond changes. This trend aligns with expectations. It confirms that the RPS method captures a wide range of chemical reactions, including those with multiple bond rearrangements. The achieved structural and reaction diversity demonstrates the potential of our method for generating datasets that enhance MLIP transferability and accuracy.

### 3.3 Performance Tests with Geometric Graph Neural Networks

Geometric GNN models are designed to capture complex interactions within molecular systems. The ultimate goal is to create a model that can reliably predict the PES of a molecule based on atomic configurations. These models process each molecule by initializing a feature vector for each atom. The chemical locality is typically encoded through layers that process interatomic distances, three‐body angles, dihedrals, or many‐body constructions. The features of each atom are refined through multiple interaction layers, and the updated feature vectors are then combined in a way to reflect the entire energy states of the molecule. In most geometric GNN models, the final energy prediction of the molecule is the sum of individual atomic contributions, reflecting the additivity principle.

Here, we report a benchmarking study of the accuracy of the MLIP created by four GNNs: SchNet,^[^ ^45^, ^46^ ^]^ PaiNN,^[^ ^47^ ^]^ NequIP,^[^ ^48^ ^]^ and MACE,^[^ ^49^ ^]^ each embodying a distinct class of neural network potentials. These models differ in their expressive powers for representing geometric (sub‐)graphs. We suggest readers refer to Duval et al.^[^ ^50^ ^]^ for a comprehensive review highlighting the evolution of MLIPs in recent years, focusing on symmetry‐based representation, general architectural choices, and interaction blocks. SchNet^[^ ^45^ ^]^ is a deep neural network based on continuous filter convolutions as our first model for comparison. It exemplifies the invariant GNNs, which ensure invariance to rotations and translations, thus respecting the fundamental symmetries of physical systems.

PaiNN^[^ ^47^ ^]^ belongs to the cartesian‐equivariant GNNs, which encode directional information and can capture anisotropic features in the data, a critical aspect when dealing with directional‐dependent physical properties. NequIP^[^ ^48^ ^]^ and MACE^[^ ^49^ ^]^ represent the spherical‐equivariant GNNs, which are designed to maintain equivariance to 3D rotations, providing a better understanding of the geometric structure. MACE^[^ ^49^ ^]^ also extends equivariant message‐passing neural networks from 2‐body to many‐body message passing in a computationally efficient manner. PaiNN, NequIP, and MACE are expected to unravel the complex landscapes of reaction mechanisms with their improved approaches to spatial relationships and symmetry, especially when confronted with the rich data procured from our reaction pathway sampling process.

**Figure 6:**
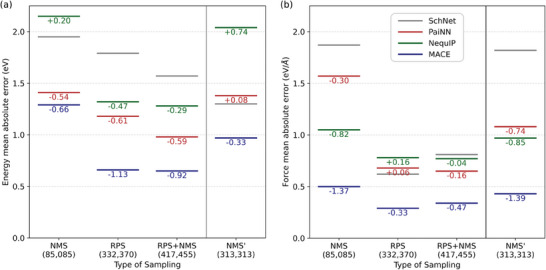
The mean absolute errors in a) energy and b) force predictions of the trained MLIP models using SchNet, PaiNN, NequIP, and MACE architectures trained with different combinations of training sets over 11,961 transition state structures from the Grambow set.^[^ ^17^ ^]^ The models were trained using datasets derived from 85 reactants: normal mode sampling (NMS, 85,085 data points), reaction path sampling (RPS, 332 370 data points), and their combination (RPS+NMS, 417,455 data points). Additionally, an expanded dataset was created using normal mode sampling from 313 reactants (NMS', 313 313 data points).

**Figure** **6** shows mean absolute errors (MAE) in energy and forces for 11 961 transition state structures from Grambow et al.,^[^ ^17^ ^]^ with all models, including SchNet, evaluated against ωB97X/6‐31G(d) QM data as the ground truth. For systematic analyses, we prepared controlled datasets to quantitatively compare the sampling effectiveness of RPS and NMS (Table S2, Supporting Information). Initially comparing datasets from identical 85 starting reactants, RPS (332 370 structures) demonstrated considerably lower prediction errors than NMS (85 085 structures). Specifically, the energy and force errors for the best model were 0.66 eV and 0.29 eV Å ^−1^ per atom, respectively. Even after expanding NMS to a comparable size (NMS': 313 313 structures from 313 reactants) by including additional reactants, RPS maintained its superior performance across all models, suggesting its fundamental advantage in sampling efficiency. While combining RPS with NMS showed modest improvements in energy prediction, force prediction accuracy remained largely unchanged. Further combination with NMS' showed similar trends (Table S2, Supporting Information).

The superior performance of RPS can be attributed to its distinct sampling characteristics. For example, the conformations sampled with RPS and NMS are visualized in Figure S4 (Supporting Information) using C_4_H_4_OCl. Meanwhile, NMS samples remain confined near the equilibrium structure, RPS effectively explores diverse reaction pathways and transition states. These results demonstrate that RPS alone efficiently captures the essential features of potential energy surfaces, particularly in the crucial transition state regions. While SchNet shows an isolated case of lower energy MAE with NMS, all models consistently favor RPS sampling for force accuracy, which we consider essential given the force‐biased nature of the loss function.

Though acceptable error thresholds can vary depending on the application, energy prediction errors ≈0.05 eV per molecule are often deemed reasonable.^[^ ^51^ ^]^ For force predictions, errors are typically expected to remain within 0.1 eV/Å per atom (Table S3, Supporting Information). Despite being trained on only 330K configurations generated from 85 initial seed molecules, our method demonstrates impressive performance, particularly in achieving high accuracy for force predictions, even in light of the inherent size‐dependence of these results.^[^ ^48^ ^]^ To estimate the transferability with respect to molecular size, we tested our best performing model (MACE) on 100 transition state structures generated by GDB9, GDB10, up to GDB13, twenty molecules from each, representing increasing complexity. Given that our toy training dataset consists of relatively small molecules (average of 10 atoms), testing sets allowed us to evaluate performance on larger molecules, up to an average of 30 atoms at GDB13. The results (Table S5, Supporting Information) demonstrate strong transferability with respect to molecular size, suggesting only a slight dependence of energy on molecular complexity.

## 4 Conclusion 

The development of accurate and transferable machine learning interatomic potentials (MLIP) has the potential to revolutionize computational chemistry by enabling large‐scale simulations with quantum‐level accuracy at a fraction of the computational cost. Our work presents an integrated approach for efficiently sampling the chemical reaction space to generate robust training data for MLIPs. The multi‐level protocol, which combines the rapid xTB method with selective high‐level DFT refinements, achieves an optimal balance between computational efficiency and accuracy. This protocol, as our main contribution, enables the generation of diverse and comprehensive datasets essential for the development of high‐performing MLIPs, and can be readily integrated into existing workflows.

Pairwise distance analysis and activation energy distributions have demonstrated the structural and reaction diversity afforded by the reaction pathway sampling (RPS) method. Unlike normal mode sampling (NMS) approaches, the RPS method captures a broader range of molecular geometries, including high‐energy transition states. This diversity is crucial for accurately describing reactive regions within the potential energy surface, thus enhancing the predictive capabilities of MLIPs. The performance evaluations reveal that GNN‐based MLIPs such as SchNet, PaiNN, NequIP, and MACE trained on RPS‐generated dataset outperform those trained on NMS dataset in terms of accuracy. These interatomic potentials have shown promise in capturing the transition state, thereby validating the efficacy of our RPS method.

Looking ahead, we identify several key directions for enhancing our approach. Although our current method effectively captures unimolecular transformations, extending it to intermolecular reactions and incorporating explicit solvent effects would significantly broaden its applicability. Furthermore, we envision improving the transition state validation process with more rigorous methods like QST,^[^ ^52^ ^]^ while maintaining our emphasis on computational efficiency. Moreover, beyond its role as a MLIP training set sampler, our method shows promise as a computational tool for rare event sampling and mechanism visualization. Future work will focus on further refining this approach and extending its application across a broader spectrum of chemical systems.

## 5 Computational Details

Driving coordinates for the SE‐GSM^[^ ^18^ ^]^ were generated by modifying the codes from Grambow et al.^[^ ^17^ ^]^ SE‐GSM calculations were conducted using pygsm^[^ ^53^, ^54^ ^]^ with GFN2‐xTB.^[^ ^22^, ^55^ ^]^ NEB^[^ ^20^, ^21^ ^]^ calculations were performed by adapting codes from Schreiner et al.^[^ ^13^ ^]^ in conjunction with the Atomic Simulation Environment.^[^ ^56^ ^]^ We executed ωB97X/6‐31G(d)^[^ ^30^, ^31^ ^]^ DFT calculations using Orca 5.0.4 version.^[^ ^57^ ^]^

We performed hyperparameter tuning for the SchNet, PaiNN, NequIP, and MACE models through manual adjustments informed by empirical results, prior experience, and standard settings from the literature. Key hyperparameters, tuning ranges, number of configurations, and other training‐related parameters for each model are detailed in Table S4 and the Computational Details for MLIP Models section of the Supporting Information.

## Conflict of Interest

The authors declare no conflict of interest.

## Author Contributions

M.L. and U.V.U. contributed equally to this work. M.L. and U.V.U. contributed to the conceptualization, methodology, software development, investigation, and drafting of the original manuscript. J.J. and I.A. were responsible for investigation. J.L. and E.S. provided conceptualization, funding acquisition, supervision, and review and editing of the manuscript.

## Supporting information

Supporting Information

## Data Availability

The data that support the findings of this study are available in the supplementary material of this article. The datasets and codes used in this work are available at https://github.com/mhyeok1/dand.
