# Supplementary material for: Automated and Efficient Sampling of Chemical Reaction Space
Source: Adv Sci (Weinh). 2025 Jan 13;12(9):2409009. doi: 10.1002/advs.202409009 (PMC11884589; doi:10.1002/advs.202409009)
Supplement: Supplementary file 1 — Supporting Information [file ADVS-12-2409009-s001.pdf]

ADVANCED SCIENCE
Open Access

Supporting Information

for *Adv. Sci.*, DOI 10.1002/advs.202409009

Automated and Efficient Sampling of Chemical Reaction Space

Minhyeok Lee, Umit V. Ucak, Jinyoung Jeong, Islambek Ashyrmamatov, Juyong Lee and Eunji Sim**

Supplementary Materials for

Automated and Efficient Sampling of Chemical Reaction Space

MINHYEOK LEE[a,†], UMIT V. UCAK[b,†], JINYOUNG JEONG[a],
ISLAMBEK ASHYRMAMATOV[c], JUYONG LEE[b,c,d,e,*], EUNJI SIM[a,*]

[a]Department of Chemistry, Yonsei University, 50 Yonsei-ro Seodaemun-gu, Seoul 03722, Korea
[b]Research Institute of Pharmaceutical Sciences, College of Pharmacy, Seoul National University, 1 Gwanak-ro, Gwanak-gu, Seoul 08832, Korea
[c]College of Pharmacy, Seoul National University, Seoul, 08826, Republic of Korea
[d]Department of Molecular Medicine and Biopharmaceutical Sciences, Graduate School of Convergence Science and Technology, Seoul National University, Seoul, 08826, Republic of Korea
[e]Arontier Co., 241, Gangnam-daero, Seocho-gu, Seoul, 06735, Republic of Korea
[†]Both authors contributed equally to this work.
*nicole@snu.ac.kr, esim@yonsei.ac.kr

Table S1: Dataset composition and size distribution across categories. All reactant structures were sourced from the GDB13 database. Categories comprise: 1234 (1-4 heavy atoms of C, N, O), 567Cl (5-7 heavy atoms with at least one Cl or S), and 8Cl (8 heavy atoms with at least one Cl or S). We used systematically designed datasets: NMS (85,085 structures from 1234 and 567Cl), RPS (332,370 structures from 1234 and 567Cl), and their combination (RPS+NMS). We further extended the comparison with NMS' (313,313 structures, including additional 8Cl data) and its combination with RPS (RPS+NMS').

	Reactant	NMS	RPS
1234	63	63,063	60,715
567Cl	22	22,022	271,655
8Cl	228	228,228	-
Total	313	313,313	332,370

Table S2: All test results over transition state structures from Grambow et al.(11,961). All models are trained on data generated by normal mode sampling (NMS) and reaction pathway sampling (RPS). We report the mean absolute error in (a) energy (eV) and (b) forces (eV/Å). Train-Validation-Test splitting is 8:1:1 after stratification.

	Energy mean absolute error (eV)					Force mean absolute error (eV/A)				
	NMS (85,085)	RPS (332,370)	RPS+NMS (417,455)	NMS' (313,313)	RPS+NMS' (645,683)	NMS (85,085)	RPS (332,370)	RPS+NMS (417,455)	NMS' (313,313)	RPS+NMS' (645,683)
SchNet	1.95	1.79	1.57	1.30	0.97	1.87	0.62	0.81	1.82	0.81
PaiNN	1.41	1.18	0.98	1.38	0.68	1.57	0.68	0.65	1.08	0.42
NequIP	2.15	1.32	1.28	2.04	1.01	1.05	0.78	0.77	0.97	0.72
MACE	1.29	0.66	0.65	0.97	0.57	0.50	0.29	0.34	0.43	0.25

Table S3: Mean absolute errors (MAE) of pre-trained models tested on 11K Grambow's transition states structures.

	Dataset size	Energy (eV)	Force (eV/$\AA$)	Level of theory
RPS sampling	330K	0.66	0.29	ωB97X/6-31G(d)
ANI-2x	8.9M conformations	0.68	0.53	ωB97X/6-31G(d)
ANI-1x	5.5M near-eq. structures	0.63	0.57	ωB97X/6-31G(d)
ANI-1ccx	10% of ANI-1x dataset	6.36	0.52	CCSD(T)/CBS
MACE-OFF23	960K SPICE DB	7.47	0.55	ωB97M-D3/def2-TZVPPD

Computational Details for MLIP models

Our tuning process focused on key parameters for each model. Specifically, for SchNet and PaiNN, we varied the number of radial basis functions (RBFs) and interaction layers within the range of 3–6. In our implementation, the SchNet and PaiNN architectures (both provided by the SchNetPack framework use a feature vector dimension of 256, comprise six interaction layers, and apply a distance cutoff of 5 Å. The radial basis for SchNet contains 300 Gaussian functions, with a grid space of the Gaussian function center of 0.2 Å. In contrast, the PaiNN model limits its radial basis functions to a maximum of 20, in alignment with DimeNet's guidelines. Throughout the training phase, the SchNet and PaiNN parameters were refined using the AdamW optimization algorithm, beginning with an initial learning rate of 0.005. The learning rate value at each epoch is dynamically adjusted by the warm restart technique implemented as the warm restart hook feature within SchNetPack.

For NequIP and MACE, we tuned the number of interaction (message passing) layers, testing 2 to 5 layers. NequIP achieved optimal performance with 5 layers, while MACE showed no improvement beyond 2 layers, so we chose this as the optimal configuration for MACE. Additionally, we experimented with the degree of spherical harmonic expansion (L) in NequIP and MACE, testing values from 1 to 4. Setting L = 4 provided only marginal gains in accuracy for energy and force, so we ultimately selected L = 3 as a balanced choice. Both models employed a cutoff radius of 5 Å were trained with a learning rate of 0.01 using an on-plateau scheduler. For all models, our loss function integrates the mean squared error for both total energy predictions and atomic force estimations, using a weighted sum to balance their contributions. We empirically determined the optimal weighting ratio between energy and force contributions to be 1:20. Training continued until convergence criteria were met, which usually required 200,000 iterations. We focused on a subset of ten configurations per model. Table S4 below summarized key hyperparameters and tuning ranges.

Table S4: Key hyperparameters, tuning ranges and number of configurations tested for each model.

Models	Hyperparameters	Values explored	Configurations tested
SchNet	Radial basis functions	20, 64, 128, 300	10 out of 48
	Interaction layers	[3-6]	
PaiNN	Radial basis functions	20, 32, 64	10 out of 36
	Interaction layers	[3-6]	
NequIP/MACE	Interaction layers	[2-5]	10 out of 48
	Spherical harmonic expansion order (L)	[1-4]	

Table S5: MACE model performance on 100 TS structures (20 structures for each database)

	GDB9	GDB10	GDB11	GDB12	GDB13
Energy (MAE eV)	0.462	0.691	0.758	0.784	1.079
Energy (MAE, eV / atom)	0.022	0.029	0.030	0.030	0.034
Force (MAE eV/Å)	0.292	0.239	0.258	0.264	0.250

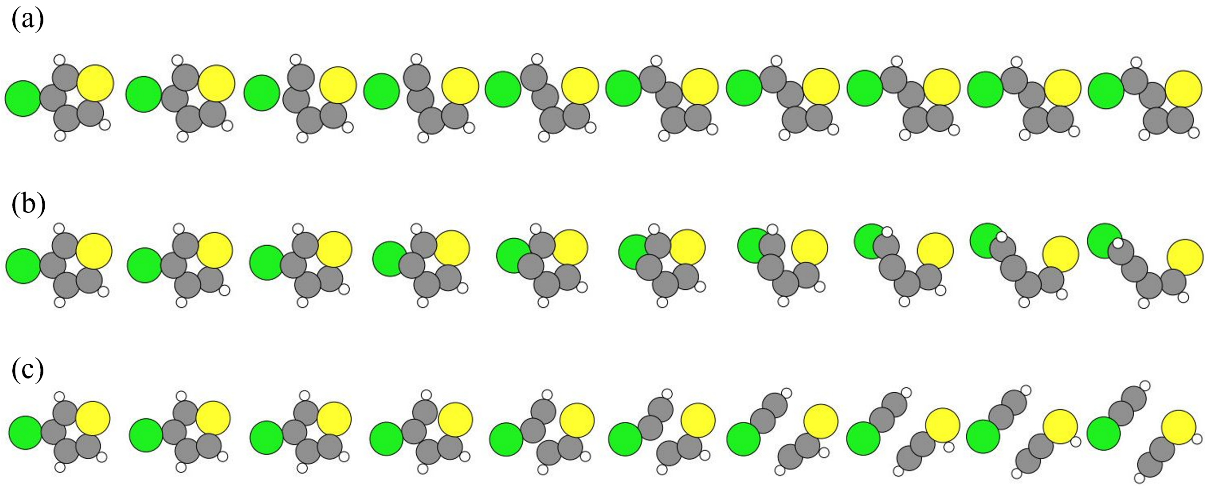

Figure S1: Examples of the reactions emanated from a single C_4H_3ClS molecule, which includes (a) ring isomerization, (b) ring breakage, and (c) fragmentation.

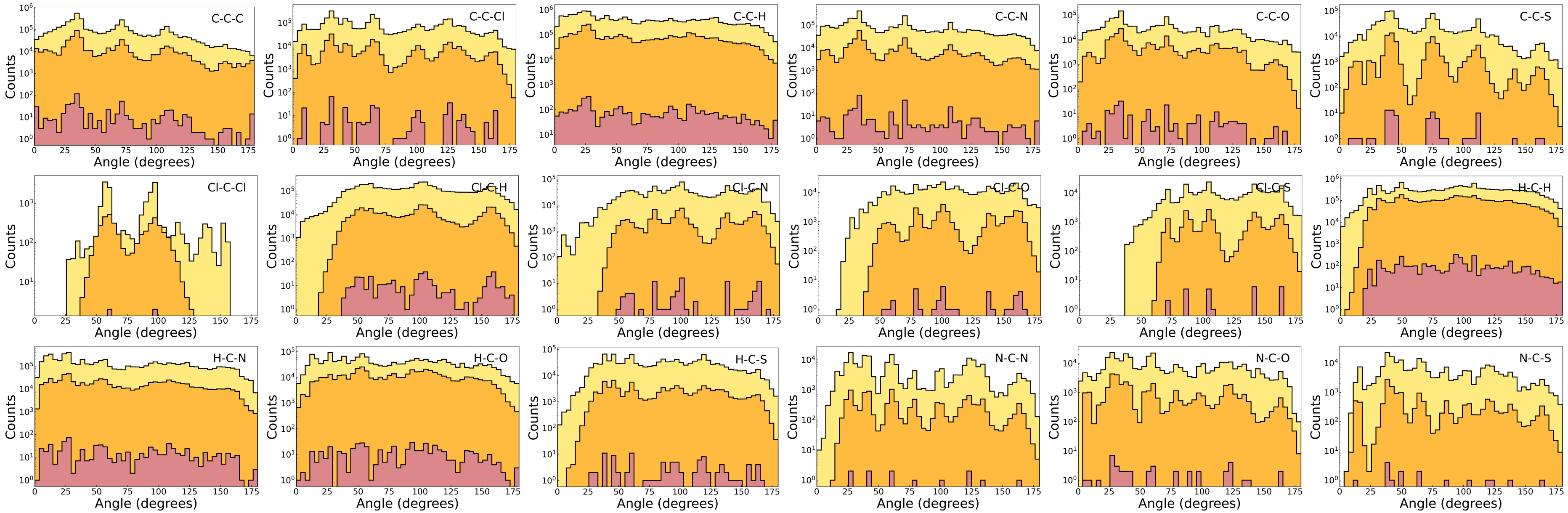

Figure S2: Enhanced structural diversity achieved by the reaction pathway sampling (RPS, yellow) method compared to normal mode sampling (NMS, orange) datasets, as illustrated by the triplets angle plots. NMS and RPS datasets originate from the same 85 equilibrium structures of reactants (Eq, red), comprising molecules with up to 4 heavy atoms (C, N, O) and those with 5-7 heavy atoms (including chlorine or sulfur).

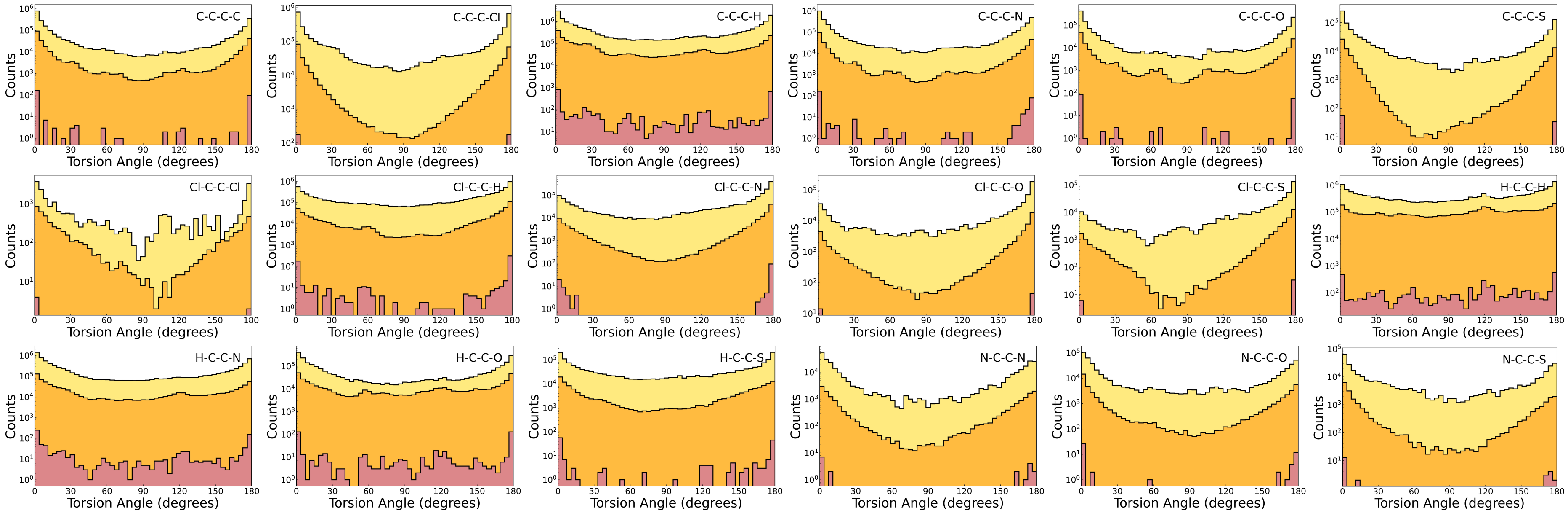

Figure S3: Enhanced structural diversity achieved by the reaction pathway sampling (RPS, yellow) method compared to normal mode sampling (NMS, orange) datasets, as illustrated by the quartet dihedral plots. NMS and RPS datasets originate from the same 85 equilibrium structures of reactants (Eq, red), comprising molecules with up to 4 heavy atoms (C, N, O) and those with 5-7 heavy atoms (including chlorine or sulfur).

(a) (b)

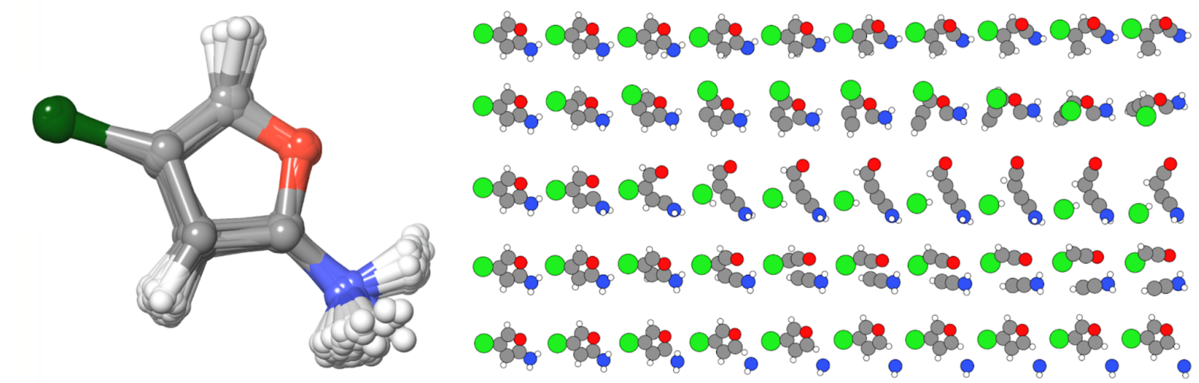

Figure S4: Comparison of sampling methods applied to C_4H_4OCl: (a) Overlay of 100 conformers generated by normal-mode sampling (b) Five distinct reaction pathways identified through our reaction pathway sampling approach.
